# Descemet's Stripping-Automated Endothelial Keratoplasty for Traumatic Aniridia and Aphakia

**DOI:** 10.1155/2012/982657

**Published:** 2012-04-09

**Authors:** Sabah S. Jastaneiah

**Affiliations:** Cornea and Anterior Segment Division, King Khaled Eye Specialist Hospital, Riyadh 11462, Saudi Arabia

## Abstract

This Interventional case reports a challenging case of descemet's stripping-automated endothelial keratoplasty (DSAEK) in a young male patient with traumatic aniridia, aphakia, and corneal edema. Surgery was planned in two stages; first was implantation of aniridia intraocular lens (AIOL), few months later, DSAEK procedure was performed. Successful outcome of both procedures was achieved as measured by the stability of the AIOL, clarity of the cornea, attachment of the lenticule, and improvement in vision. Aniridia implant supports a sufficient amount of air in the anterior chamber especially if the posterior segment is well formed, while providing the required lens power to improve vision. DSAEK procedure challenges that include iris defects and aphakia may be overcome by stepwise planning of the procedure.

## 1. Introduction

Descemet stripping automated endothelial keratoplasty (DSAEK) is a procedure used for target replacement of a dysfunctional endothelial cell layer [1]. It has many advantages over conventional penetrating keratoplasty (PKP) in terms of faster visual rehabilitation, induced refractive error (refractive neutral), minimal ocular surface-related changes including sutures and surface-keratopathy-related complications [1, 2]. One of the most important advantages of this procedure over conventional keratoplasty is the maintenance of the structural integrity of the eye, especially in the younger patients where trauma is a higher possibility [3].

Surgically challenging cases have been described in the literature including cases with Iridocorneal Endothelial Syndrome (ICE) [4], aniridia and aphakia [5], complex anterior chambers with anterior chamber lenses [6, 7], post keratoplasty [8], and pediatric patients [5, 9–11].

The aniridia intraocular lenses or the iris reconstruction intraocular lenses can be used to correct congenital and traumatic aniridia. These lenses are designed for scleral fixation or sulcus fixation, depending on the clinical condition of the zonulae and the sufficiency of the capsular support. Indications of the implants are aniridia or iris coloboma to eliminate glare and control the amount of light that enters the eye. These lenses can provide additional optical correction and they come in different sizes, shapes, and color (rings or implants).

Patients with combined traumatic aniridia, aphakia, and endothelial failure can enjoy the advantages of the DSAEK procedure in addition to the benefits of an implantable aniridia intraocular lens (IOL).

Here, I describe a stepwise approach to manage a case of a young patient having traumatic aniridia, aphakia and corneal edema.

## 2. Case Presentation

**Figure 1:**
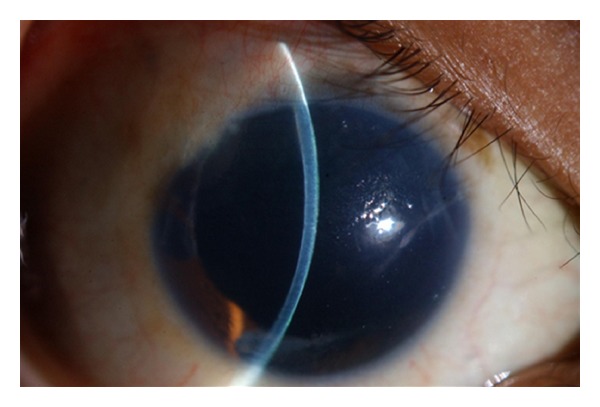
Clinical picture on presentation showing corneal edema, peripheral corneal scarring, aniridia with only a sector of iris tissue remaining temporally, and aphakia.

After approval of the human ethics committee/Institutional Review Board, this case describes a young male patient who had trauma to his right eye with a knife 2 years prior to his presentation. At the time of trauma, he had primary repair of the corneal laceration with lensectomy and anterior vitrectomy. The patient was referred to our facility with corneal edema, angle recession glaucoma, traumatic aniridia, and aphakia, for further management. Corneal thickness was in the range of 700 microns with reasonable view to the anterior segment. Uncorrected visual acuity was 20/200. Good visual potential of 20/40 was achieved with contact lenses, but the patient could not tolerate contact lens wear (Figure 1).

A two-stage management plan was elected in the form of aniridia intraocular lens (AIOL) implantation followed by DSAEK. The main objective was to give him a chance to have the best long-term outcome to his corneal graft in addition to the benefit of the aniridia implant.

### 2.1. Aniridia Implant Procedure

**Figure 2:**
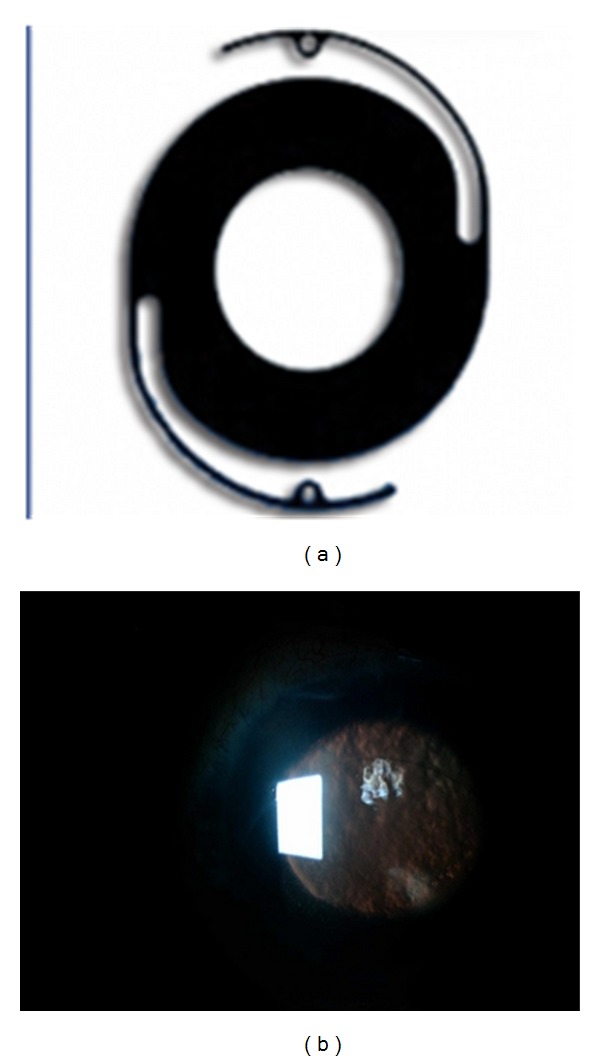
(a) Morcher aniridia Intraocular Lens of +22 diopters and a 5 mm pupil zone. (b) Clinical picture using retro illumination after the aniridia IOL implantation demonstrating the jet black reflection out side the central optical zone and corneal edema.

The first procedure was done in April 2008. A scleral-fixed Morcher AIOL (Figure 2(a)) of +22.0 D power, model 67G, and 5 mm pupil zone was implanted under general anesthesia. Patients own keratometry reading was used for the IOL calculations, with an intended under correction to achieve a target of −2 diaopteric power. This is in order to overcome the hyperopic shift after the DSAEK procedure (Figure 2(b)).

### 2.2. DSAEK Procedure

Six months later, DSAEK was performed. The procedure started by preparing the host cornea. Methylcellulose as an ophthalmic viscoelastic devise (OVD) was injected into the anterior chamber (AC) through a peripheral paracentesis. Recipient's descemet's membrane was peeled out of the eye through a 3 mm clear cornea temporal incision performed using a diamond blade. The viscoelastic material was washed out of the anterior chamber using an irrigation aspiration technique after enlarging the corneal wound to 4 mm.

The donor cornea lenticule was prepared using an artificial anterior chamber and an automated Moria microkeratome.

The donor lenticule that includes the descemet's membrane and part of the posterior stroma was folded in a taco fashion and inserted into the recipient eye. After implantation, air was injected in the anterior chamber and high pressure of around 40 mmHg was maintained for 8 minutes. Intraoperatively, the air bubble was maintained in the AC and the donor cornea lenticule was in place, but once air was replaced by balanced salt solution (BSS), the globe softened. Intraoperative challenges were mainly related to hypotony although the corneal wound was well secured with interrupted 10.0 nylon sutures.

At the end of the procedure, 30–40% of the air was kept in the AC and the patient was instructed to be in supine position.

One hour after the procedure, the patient was checked in the holding area, finding a detached lenticule, he was taken to an operative microscope and air was injected, it was noticed that it was seeping to the posterior segment through the peripheral part of the aniridia implant but the IOP was stable as this was a vitrectomized eye.

Next day, he had corneal edema, partial attachment of the inferior part of the lenticule, slight inferior decenteration with 30% air in the AC. The eye was soft, measuring about 8 mmHg. Air was reinjected under slit-lamp, using an aseptic technique, in the examination room, and the slightly inferiorly decentered lenticule was gently pushed to the center. An interesting observation was noticed while intracameral air was injected: part of the air was inadvertently seeping under the implant, which helped in firming the globe, while the rest was maintained in the AC securing the lenticule.

On the second postoperative day, he had the same condition of a partially detached, inferiorly decentered, edematous lenticule, and corneal stroma edema. It was elected to observe the cornea for 24 hours before rebubbling; accordingly, no intervention was done. The eye was firmer with normal intraocular pressure of 14 mmHg. The plan was to rebubble and center the lenticule if no improvement was documented after 24 hours.

The patient was examined early morning on the third postoperative day before the planned procedure. Clinically, he was found to have a spontaneously attached slightly decentered lenticule with clear cornea. The rest of the clinical examination included a normal IOP, deep AC, AIOL in good position, and about 10% air in the AC.

**Figure 3:**
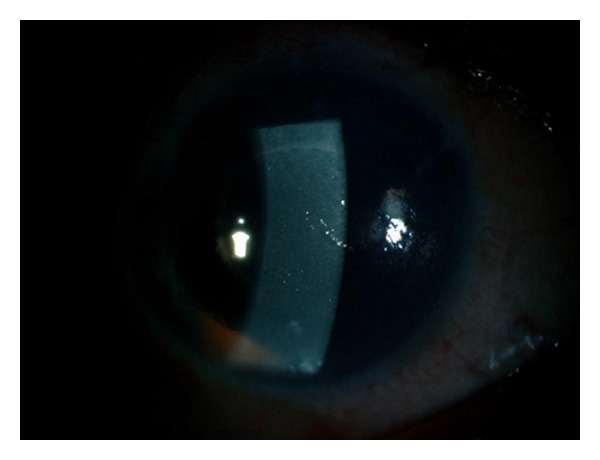
Broad slit lamb photograph showing clear slightly decentered corneal graft, the aniridia optical zone area surrounded by a black diaphragm of the aniridia implant.

Last followup was on February 2010; uncorrected vision improved to 20/40, controlled IOP with topical antiglucoma medications and clear cornea with slightly decentered lenticule (Figure 3).

## 3. Discussion

Patients having aniridia or any iris defects, aphakia, and corneal decompensation were considered a relative contraindication to undergo DSAEK, and they were deprived of the many advantages of endothelial keratoplasty.

Many of these patients are young and are more vulnerable to trauma; performing penetrating keratoplasty on their eyes further weakens the structural integrity of the eye making any minor insult major and may end up with a devastating outcome.

Performing DSAEK in these cases is a true surgical challenge; the main concerns are the risk of dropping the donor corneal lenticule, dropping of pieces of the recipient's corneal descemet's membrane, and the difficulty in maintaining the air bubble intended to push the donor corneal button against the recipient's host cornea.

In this case, we had different management options that included conventional penetrating keratoplasty with secondary scleral-fixed IOL or performing the DSAEK procedure alone as have been described previously by Price et al. [5]. I elected to go for a stepwise approach performing the sclera-fixed aniridia implant first then performing the DSAEK procedure. This way, I would avoid having any other intraocular procedure after the DSAEK that may compromise the endothelial cell count and function. The procedure could have been performed simultaneously by implanting the AIOL and performing the DSAEK at the same time. But since I have done this before and faced the challenge of a large wound with less firm globe and difficulty in maintaining air in the AC, I preferred to go for this stepwise approach especially that the cornea, although decompensated, allowed for a good intraoperative view.

Both procedures were performed in a convenient way without major difficulties. The only challenge was having the IOP to the norm of the high side during the DSAEK procedure, even though the wound was constructed to be self-sealing. Additional sutures were placed at the incision site, and steady inflation of the eye was achieved by alternating injections of air and BSS solution in the AC. In vitrectomized eyes, additional amount of air is required to be injected intracamerally in order to have a nice firm globe. Allowing part of that air to seep behind the IOL will help in achieving the required firm globe, but without overfilling the posterior chamber to avoid iris or IOL forward movement. Another possible intraoperative challenge would have been slipping of the lenticule to the posterior segment that could happen through the space between the AIOL and the angle.

Postoperatively, the donor lenticule was partially attached until the eye developed normal IOP and was firm enough to support its complete attachment to the back of the recipient's stroma even without the need to rebubble. Additionally, it was observed that the lenticule has repeated tendency to move away from the previously deep traumatic corneal scar despite being repositioned twice centrally on the day of surgery and the first postoperative day. This may be related to posterior corneal excretions preventing the attachment of the lenticule to that area; hence the lenticule decentered to be attached to the smooth posterior corneal surface. Learning this retrospectively; I would advise placing an anchoring full thickness corneal suture to the area of concern.

Lessons that I learnt from this case were many; including the advantages of step-by-step planning of challenging procedures, the efficacy of the aniridia implant to support a good amount of air intra- and postoperatively, the importance of having a firm eye to aid the donor lenticule attachment especially in previously vitrectomized eyes, and finally the possibility of spontaneous attachment of the donor lenticule within a period of 2-3 days especially if it was partially attached and there was no obvious anatomical reason for it not to attach.

In conclusion, DSAEK procedure challenges that include iris defects and aphakia may be overcome by stepwise procedure planning. In addition to the known benefits of the aniridia implant, it supports a reasonable amount of air in the anterior chamber especially if the posterior segment is well formed.

Spontaneous attachment of the DSAEK lenticule is a possibility that should be considered before rebubbling or judging the graft as failed to attach. The average time period of the attachment in this case was at the third postoperative day.

## References

[1] Melles GRJ, Lander F, Rietveld FJR (2002). Transplantation of Descemet’s membrane carrying viable endothelium through a small scleral incision. *Cornea*.

[2] Price FW, Price MO (2005). Descemet’s stripping with endothelial keratoplasty in 50 eyes: a refractive neutral corneal transplant. *Journal of Refractive Surgery*.

[3] Elder MJ, Stack RR (2004). Globe rupture following penetrating keratoplasty: how often, why, and what can we do to prevent it?. *Cornea*.

[4] Price MO, Price FW (2007). Descemet stripping with endothelial keratoplasty for treatment of iridocorneal endothelial syndrome. *Cornea*.

[5] Price MO, Price FW, Trespalacios R (2007). Endothelial keratoplasty technique for aniridic aphakic eyes. *Journal of Cataract and Refractive Surgery*.

[6] Wylegała E, Tarnawska D (2008). Management of pseudophakic bullous keratopathy by combined Descemet-stripping endothelial keratoplasty and intraocular lens exchange. *Journal of Cataract and Refractive Surgery*.

[7] Lake DB, Rostron CK (2008). Management of angle-supported intraocular lens and iridectomy in descemet-stripping endothelial keratoplasty. *Cornea*.

[8] Price FW, Price MO (2006). Endothelial keratoplasty to restore clarity to a failed penetrating graft. *Cornea*.

[9] Jeng BH, Marcotty A, Traboulsi EI (2008). Descemet stripping automated endothelial keratoplasty in a 2-year-old child. *Journal of AAPOS*.

[10] Ponchel C, Malecaze F, Arné JL, Fournié P (2009). Descemet stripping automated endothelial keratoplasty in a child with descemet membrane breaks after forceps delivery. *Cornea*.

[11] Ghaznawi N, Chen ES (2010). Descemet’s stripping automated endothelial keratoplasty: innovations in surgical technique. *Current Opinion in Ophthalmology*.

